# Ethylene Role in Plant Growth, Development and Senescence: Interaction with Other Phytohormones

**DOI:** 10.3389/fpls.2017.00475

**Published:** 2017-04-04

**Authors:** Noushina Iqbal, Nafees A. Khan, Antonio Ferrante, Alice Trivellini, Alessandra Francini, M. I. R. Khan

**Affiliations:** ^1^Department of Botany, Jamia HamdardNew Delhi, India; ^2^Plant Physiology and Biochemistry Laboratory, Department of Botany, Aligarh Muslim UniversityAligarh, India; ^3^Department of Agricultural and Environmental Sciences, Università degli Studi di MilanoMilano, Italy; ^4^Institute of Life Sciences, Scuola Superiore Sant’AnnaPisa, Italy; ^5^Crop and Environmental Sciences Division, International Rice Research InstituteManila, Philippines

**Keywords:** ethylene, flower senescence, fruit ripening, leaf senescence, phytohormones, VOCs

## Abstract

The complex juvenile/maturity transition during a plant’s life cycle includes growth, reproduction, and senescence of its fundamental organs: leaves, flowers, and fruits. Growth and senescence of leaves, flowers, and fruits involve several genetic networks where the phytohormone ethylene plays a key role, together with other hormones, integrating different signals and allowing the onset of conditions favorable for stage progression, reproductive success and organ longevity. Changes in ethylene level, its perception, and the hormonal crosstalk directly or indirectly regulate the lifespan of plants. The present review focused on ethylene’s role in the development and senescence processes in leaves, flowers and fruits, paying special attention to the complex networks of ethylene crosstalk with other hormones. Moreover, aspects with limited information have been highlighted for future research, extending our understanding on the importance of ethylene during growth and senescence and boosting future research with the aim to improve the qualitative and quantitative traits of crops.

## Introduction

The growth and development of plants under varied environmental conditions determine agricultural production. The growth, development, and senescence of plant’s organs can influence crop production by modulating photosynthesis, nutrient remobilization efficiency, and harvest index (Paltridge et al., 1984; Jing et al., 2005; Iqbal et al., 2012). Phytohormones have been shown to increase growth and yield of plants. The phytohormone ethylene controls growth and senescence of plants (Reid, 1995; Lutts et al., 1996; Thompson et al., 1998; Pierik et al., 2006; Masood et al., 2012; Nazar et al., 2014). Ethylene is regarded as a multifunctional phytohormone that regulates both growth, and senescence. It promotes or inhibits growth and senescence processes depending on its concentration, timing of application, and the plant species. The application of ethephon, an ethylene releasing compound enhanced ethylene evolution and increased leaf area of mustard at a lower concentration, while inhibited at higher concentration (Khan, 2005; Khan et al., 2008).

Ethylene governs the development of leaves, flowers, and fruits. It may also promote, inhibit or induce senescence depending upon the optimal or sub-optimal ethylene levels (Konings and Jackson, 1979; Khan, 2005; Pierik et al., 2006). It appears quite interesting to examine how the same hormone influences the two contradictory processes of growth and senescence. This review covers the discussion on the role of ethylene in the growth and development and explores its interaction with other hormones in regulating these processes.

## Leaf Growth and Development

### Ethylene

Leaf growth and development are affected by various environmental factors and endogenous hormonal signals (**Table 1**). These processes are regulated by phytohormones, transcriptional regulators and mechanical properties of the tissue (Bar and Ori, 2014). The role of ethylene in the leaf growth and development has been confirmed physiologically using ethylene inhibitors, and genetically using ethylene-insensitive mutants or transgenic plants lacking the key enzymes of ethylene biosynthesis (Oh et al., 1997; Bleecker et al., 1998). It has been observed that ETHYLENE RESPONSE FACTOR5 (ERF5) and ERF6, in *Arabidopsis*, improve leaf growth to environmental challenges (Dubois et al., 2015). The response of leaf growth and development to ethylene depends on concentration and species involved in the study (Fiorani et al., 2002; Kawa-Miszczak et al., 2003; Khan, 2005). In support of this, Fiorani et al. (2002) showed that the slower growing species of *Poa* (*Poa alpina* and *Poa compressa*) were more responsive to ethylene, with greater inhibition in leaf elongation than the fast growing species. However, a promoting effect on leaf elongation rate at a low ethylene concentration was observed in the slower growing species, while at the same concentration, leaf elongation rate was only slightly inhibited in the two fast-growing species. This response was reversed at higher concentrations, showing an inhibition effect. The study of Khan (2005) on mustard suggested that there exists a correlation between ethylene and growth of plants following the defoliation of mature leaves. Furthermore, ethylene-insensitive genotypes of *Arabidopsis* (*Arabidopsis thaliana*), tobacco (*Nicotiana tabacum*) and petunia (*Petunia x hybrid*) showed no increase in the total leaf area when compared to normal ethylene-sensitive control plants (Tholen et al., 2004). Treatment with ethephon, a compound that releases ethylene, resulted in an increase in both ethylene biosynthesis and leaf area expansion (Khan, 2005; Khan et al., 2008). In contrast, Voisin et al. (2006) determined that the rate of ethylene evolution had no relation to the leaf elongation rate variability in maize (*Zea mays*). Ethylene-induced reductions in leaf growth have been reported in pea (*Pisum sativum*) plants, around which rhizobacteria with enhanced ACC deaminase activity were added to soil (Belimov et al., 2009). Interestingly, the lower leaf area was observed in lettuce (*Lactuca sativa*) grown in closed environments, where ethylene produced from the plants reached stressful levels. The plants showed lower relative leaf growth rates compared to those grown in containers from which the ethylene had been scrubbed (He et al., 2009). This reduced leaf area was an indirect effect of ethylene on the leaf epinasty with reduced light capture, and/or on a reduced CO_2_ assimilation, which was found to be more sensitive to the ethylene increase than the reduction in growth. Both reactive oxygen species (ROS) and nitric oxide (NO), which could be potentially up-regulated by ethylene, have also been involved in leaf expansion (Wilkinson and Davies, 2010).

**Table 1:** Some representative studies in relation to the effect of ethylene on leaf growth, development and senescence.

Plant Species	Ethylene treatments	Effect on plants	Reference
Cotton (*Gossypium hirsutum*)	12 μL L^-1^ of ethylene at 18 l min^-1^	Leaf abscission in plants 5 weeks olds	Beyer, 1976
Rice (*Oryza sativa*)	Submergence condition (7 days) with 1 μL L^-1^ in the internods	Stimulate the internodes growth 3–5 fold	Métraux and Kende, 1983
Celery-leaved buttercup *(Ranunculus sceleratus)*	10–50 nL mL^-1^ ethylene	Increased 4–5 times petiole growth	Musgrave and Walters, 1973
Maize (*Zea mays*)	0.1–5.0 μL L^-1^ ethylene	Inhibited leaf extension	Jackson et al., 1981
*Arabidopsis* (*Arabidopsis thaliana*)	Col-0 plants exposed to 1–5 μL L^-1^ ethylene	Strong hyponastic response of leaf	Millenaar et al., 2005
Oat (*Avena sativa*)	0.2 Mm ACC	Leaf chlorophyll loss followed by leaf senescence.	Gepstein and Thimman, 1987
Potato (*Solanum tuberosum*)	5 nL L^-1^	Severe leaf senescence symptoms such as yellowing, epinasty and lack of growth	Özgen et al., 2005
Rocket salad (*Eruca sativa*)	1 μL L^-1^	Leaf chlorophyll loss	Koukounaras et al., 2006

The ethylene effect on leaf growth and development may be independent or dependent on its interaction with other hormones. Multiple receptors of one phytohormone might be involved in non-redundant responses, either in different tissues, at different developmental stages, or upon different environmental cues.

### Interaction between Ethylene and Other Hormones during Leaf Growth and Development

The following section highlights the interaction of ethylene with other hormones and plant responses during leaf growth and development.

#### Ethylene and Auxin

The action of ethylene on leaf growth may be auxin-dependent or auxin-independent. Hormonal coordination is an important aspect, which regulates leaf growth processes. Auxin induces ethylene production, and many effects of exogenous auxins are, in fact, ethylene responses (Abeles et al., 1992). Auxin plays an important role in leaf development and it is potentially able to increase shoot apical meristem at the time of leaf initiation, through increased auxin biosynthesis (Cheng et al., 2007; Pinon et al., 2013). Auxin regulates the initiation of the new leaves in tomato (*Solanum lycopersicum*) (Reinhardt et al., 2000), leaf vascular development (Mattsson et al., 2003), and cell division phase during leaf expansion (Ljung et al., 2001) in *Arabidopsis*. In *Arabidopsis*’s shoot, the radial position and the size of the leaf during organ formation were mediated by indole-3-acetic acid (IAA) (Reinhardt et al., 2000).

Studies have shown that leaf epinasty could be attributed to auxin-stimulated ethylene or an activity of auxin alone (Abeles et al., 1992; Sterling and Hall, 1997; Grossmann, 1998; Hansen and Grossmann, 2000). The inhibition of leaf growth induced by auxin was found to be independent of ethylene in common bean (*Phaseolus vulgaris*) plants (Keller et al., 2004). The inhibition of ethylene by applying 1 mM ethylene synthesis inhibitor aminooxyacetic acid (AOA) with 1 mM IAA did not affect auxin-induced inhibition of leaf growth. The daily application of 1 mM AOA alone did not affect leaf growth, but over a 24-h period, a 1 mM AOA treatment was effective as an inhibitor of ethylene biosynthesis in detached common bean leaves.

#### Ethylene and Cytokinin

The reports on the interaction between ethylene and cytokinin are scanty. However, cytokinin may play an important role in leaf initiation through the maintenance of S-adenosyl-methionine (SAM); an immediate metabolite for biosynthesis of ethylene (Kurakawa et al., 2007; Gordon et al., 2009). Cytokinins regulate a wide range of growth and developmental processes throughout the life cycle of a plant, including seed germination, leaf expansion, induction of flowering, as well as flowering and seed development (Sun and Gubler, 2004; Yamaguchi, 2008). In Zea mays, the response regulator (RR) protein ABPHYL1 (ABPH1), together with PIN1, was also expressed at the site of future leaf initiation, and both are induced by the cytokinins (Lee et al., 2009). ABPH1 positively regulates organ initiation, perhaps by inhibiting the cytokinins response. Carabelli et al. (2007) showed that CKX6, a cytokinin oxidase/hydrolase, was induced in the simulated shade and promoted cytokinin depletion specifically in pre-procambial cells of developing leaf primordial, substantiating the role of cytokinin breakdown in the inhibition of leaf development during shade avoidance. The CKX6 induction has been reported to be an auxin response and was mediated by the auxin receptor TIR1. Thus, auxins and cytokinins act synergistically in leaf development. However, more efforts should be done to gain a better understanding on the crosstalk of ethylene with cytokinins in the whole context especially on the link between SAM and cytokinins regulation.

#### Ethylene and Gibberellins

Gibberellins (GA) play a role in the leaf expansion. Spraying dilute aqueous GA solutions to leaves and stems of potato caused lesser internode growth with larger leaf growth and mature leaves (Humphries, 1958). The response of GA on the leaf development showed photoperiodic regulation of the leaf elongation in bluegrass (*Poa pratensis*), and indicated a photoperiodic control of oxidation of GA_53_ to GA_44_ and GA_19_ to GA_20_, and also of 3 β-hydroxylation of GA_20_ to GA_1_ (Junttila et al., 1997). Limited water sources can also cause reductions in leaf size (Sack et al., 2003; Royer et al., 2005). In *Arabidopsis*, these responses were at least in part executed by ethylene response factors and GA catabolism (Dubois et al., 2013).

The crosstalk between ethylene and GA was revealed from the study of Achard et al. (2006), who found that the destabilization of DELLA proteins induced by GA was modulated by environmental signals and the plant hormone signaling (such as auxin and ethylene). De Grauwe et al. (2008) reported that a functional GA response pathway was required for the increased ethylene biosynthesis in eto2-1 (ethylene overproducing mutant) since the gai eto2-1 (GA-insensitive; ethylene overproduction) double mutant did not overproduce ethylene, suggesting that the stability of the ACS5 protein was dependent on GA. The GA-signaling cascade appeared to be regulated by ethylene in GIBBERELLIN INSENSITIVE DWARF1 (GID1c) (more than twofold induction after ethylene treatment) (Zimmermann et al., 2004; Dugardeyn et al., 2008). The relationship between ethylene signaling and the GA–GID1–DELLA mechanism has shown that ethylene inhibited the seedling growth DELLA-deficient mutant Arabidopsis less than wild type. However, ethylene inhibited the GA-induced disappearance of GFP-RGA via CTR1-dependent signaling (Achard et al., 2003), indicating an antagonistic relation between ethylene and GA. Dubois et al. (2013) reported that under osmotic stress in actively growing leaves, the expression of ERF5 and ERF6 gene was induced. The expression of ERF6 gene inhibited the cell proliferation and growth of leaves and the inhibition was dependent on gibberellin and DELLA signaling. ERF6 provides a link between 1- aminocyclopropane 1 carboxylic acid (ACC), the ethylene forming enzyme and DELLA signaling in the cell cycle pause-and-stop model, improving our understanding of growth inhibition in the proliferating leaf primordia of plants subjected to water limitation.

#### Ethylene and Abscisic Acid

Abscisic acid (ABA) may limit ethylene production to enhance leaf growth (Hussain et al., 2000). Normal levels of endogenous ABA maintains leaf expansion in *Arabidopsis*, partly through limiting ethylene biosynthesis and partly by another mechanism that is independent of ethylene. To analyze how ABA functions by ethylene suppression, LeNoble et al. (2004) studied ABA-deficient (*aba2-1*) and ethylene-insensitive (*etr1-1*) single and double mutants of *Arabidopsis*. Shoot growth was found to be inhibited in ABA-deficient *Arabidopsis*. Exogenous treatment with ABA resulted in the complete recovery of shoot growth in *aba2-1* relative to wild type, and also significantly increases growth of *aba2-1 etr1-1.* The total leaf area and shoot fresh weight were not significantly lower than in *etr1-1*. ABA from avocado (*Persea americana*) induced formation of “floating-type” leaves in American pondweed (*Potamogetgon nodosus*) at low concentration (5 × 10^-7^ M) (Anderson, 1982). The influence of the ABA on the leaf growth could be through increased conductance to water transfer in plants as a result of an increased tissue hydraulic conductivity (Tardieu et al., 2010).

## Leaf Senescence

### Ethylene

Ethylene has an important role in the regulation of leaf senescence. Ethylene is one of the most important hormones in the leaf senescence regulation (**Table 1**). Ethylene can trigger the senescence process, especially in the sensitive species. The ethylene biosynthesis is higher during the first stage of leaf formation and declines until it reaches maturity when the leaf is completely expanded, then it increases again during the early step of the senescence initiation. The ACC content only increases in senescing leaves and shows the same pattern of ethylene production (Hunter et al., 1999). At the molecular level, it has been shown that different genes of the same family encode for the enzymes of ethylene biosynthesis that are activated during leaf development and their expression is timely regulated (Hunter et al., 1999). The biosynthesis occurs in any part of the plant and at any stage of leaf development. Consequently, the biological responses depend on the tissue sensitivity. The exposure of plant sensitive to ethylene induces premature senescence symptoms such as leaf yellowing, abscission, or desiccation/necrosis. The plant responses to ethylene vary considerably between and within species and are modulated by differential hormonal sensitivity. The visual symptoms of leaf senescence are represented by chlorophyll degradation and the leaf abscission (Lewington et al., 1967; Gepstein and Thimman, 1987). At the molecular level, ethylene has been shown to be involved in the organized cell dismantling and the activation of nutrients recycling from senescing leaves to the other organs. Leaf cells undergo a sequential and organized dismantling process which includes nucleic acid reduction, protein degradation, and turnover reduction, (Lutts et al., 1996), membrane disruption, lipid degradation, peroxidation (Buchanan-Wollaston, 1997; Thompson et al., 1998; Buchanan-Wollaston et al., 2003), and leaf pigment breakdown (Matile et al., 1996).

Leaf senescence is activated at the mature stage of leaf development when leaves are fully expanded. During leaf senescence, three different stages can be identified: initiation, organization of degradation, and death processes.

The most common visible symptom of leaf senescence is the yellowing caused by the chlorophyll degradation and its impaired biosynthesis. The initial step of chlorophyll breakdown is catalyzed by chlorophyllase that convert chlorophyll *a* and *b* to chlorophyllide and phytol (Matile et al., 1996). Chlorophyll loss increases after ethylene exposure in different cut flowers such as stock (*Matthiola incana*) and chrysanthemum (*Dendranthema grandiflora*; Reyes-Arribas et al., 2001) and many other flowers (Ferrante et al., 2004, 2005, 2009). Özgen et al. (2005) have reported that the signs of senescence induced by ethylene include malformed, thickened leaves, lack of growth and epinasty. Tobacco leaves treated for 24 h showed higher chlorophyll degradation but did not anticipate the increase in ethylene biosynthesis and respiration, typically of the climacteric trend (Aharoni and Lieberman, 1979). The effect of ethylene was found tightly associated with leaf age as demonstrated in old *Arabidopsis* mutants and also depended on the length of the treatment (Jing et al., 2005). The chlorophyll reduction has also been observed in rocket salad (*Eruca sativa*) leaves exposed to 1 μL L^-1^ during storage, a condition that shortens the shelf life by approximately 2 days (Koukounaras et al., 2006). Another effect induced by ethylene on leaf senescence is the abscission or induction of necrosis. Leaf abscission is a coordinated process, which involves several structural changes in the cells located in the abscission zone. It is a seasonal process that normally occurs in deciduous plants (Taylor and Whitelaw, 2001), where ethylene and auxins also have a crucial role.

### Interaction between Ethylene and Other Hormones during Leaf Senescence

Plant hormones can repress or enhance leaf senescence in plants or after harvest. This section gives an insight into the interaction of ethylene with other hormones and provides responses during leaf senescence.

#### Ethylene and Auxin

Leaf senescence is affected by auxin content and ethylene biosynthesis (Ferrante and Francini, 2006). In particular, leaf abscission is under the control of auxin and ethylene. Burg (1968) suggested that ethylene caused leaf abscission *in vivo* by inhibiting auxin synthesis and transport or enhancing auxin degradation, thus, lowering diffusible auxin level. In the abscission zone, ethylene and auxin act antagonistically and auxin concentrations were associated with tissue sensitivity to ethylene. The equilibrium between ethylene and auxin is crucial for the regulation of leaf abscission. During leaf senescence, the auxin concentration declined and tissue sensitivity to ethylene increased as well as ethylene biosynthesis (Brown, 1997). Using a transcriptome approach, 1,088 transcription factors (TFs) were found to be differentially regulated in the soybean leaf abscission. Among these, 188 TFs were differentially expressed in the abscission zone (Kim et al., 2016). Ethylene and auxin were strongly regulated by these transcription factors. However, the exogenous applications of these hormones also regulated the expression of these genes delaying or anticipating the leaf senescence and abscission. Riov and Goren (1979) suggested that ethylene inhibited auxin transport in the veinal tissues and reduced the amount of auxin transported from the leaf blade to the abscission zone in orange (*Citrus sinensis*), necklace poplar (*Populus deltoids*), and eucalyptus (*Eucalyptus camaldulensis*). La Rue (1936) showed that the removal of leaf blade induced abscission, but the application of auxin to the site of removal resulted in the inhibition of abscission. Ethylene has been shown to play an antagonistic role to auxins in the abscission of various organs. Abscission was delayed in the ethylene-insensitive *Arabidopsis* mutants *ein2* and *etr1-1* (Patterson and Bleecker, 2004), while ethylene application hastened abscission in various organs and species. Ethylene induced the expression of a polygalacturonase which is required for cell separation in tomato petioles (Hong et al., 2000; Jiang et al., 2008) and interestingly this polygalacturonase was inhibited by the exogenous auxin. This suggested the antagonistic effects of auxin and ethylene in the abscission.

#### Ethylene and Cytokinins

Cytokinins can suppress leaf senescence leading to greater retention of chlorophyll known as Richmond and Lang (1957) demonstrated. The effect of cytokinins on leaf senescence was demonstrated by the autoregulation of cytokinins biosynthesis during senescence using an isophentenyl transferase (*IPT*) gene under the regulation of senescence-associated gene 12 (*SAG12*) promoter (Gan and Amasino, 1995). This promoter has been widely used to activate genes expression during senescence. The SAG12 gene encodes for a cysteine protease that was activated during senescence independently from the trigger events. Therefore, the *SAG12* promoter can have great application in the agricultural science and the postharvest sector. Deletion studies on the *SAG12* promoter demonstrated that young and mature leaves contained factors that exhibited differential binding to the senescence responsive promoter element (Noh and Amasino, 1999). The construct *SAG12::IPT* gene has been studied in different species and all showed delayed senescence. This strategy was effective in delaying leaf senescence in several crops such as alfalfa (*Medicago sativa*; Calderini et al., 2007), broccoli (*Brassica oleracea*; Chen et al., 2001), cassava (*Manihot esculenta*; Zhang et al., 2010), creeping bentgrass (*Agrostis stolonifera*; Xu et al., 2009), lettuce (McCabe et al., 2001), petunia (Chang et al., 2003), rose (*Rosa hybrid*; Zakizadeh et al., 2013), tobacco (Jordi et al., 2000), and wheat (*Triticum aestivum*; Sýkorová, 2008). The senescence delay reduced ethylene biosynthesis in the transformed plants. The exogenous application of cytokinins in potted and cut flowers delayed the leaf yellowing and decreased ethylene biosynthesis. The 6-benzyladenine (BA) applied as pulse treatments successfully delayed leaf yellowing in cut goldenrod (*Solidago canadensis*; Philosoph-Hadas et al., 1996), potted lilies (*Lilium longiflorum*; Han, 1997), and cut Peruvian lily (*Alstreomeria*) flowers (Mutui et al., 2003). The effect of BA treatment on the ethylene is due to the inhibition of leaf senescence that leads to lower ethylene biosynthesis.

#### Ethylene and Gibberellins

Gibberellins are considered as leaf senescence inhibitors and are able to avoid or delay leaf yellowing. Gibberellins are commonly used as postharvest treatments in several cut flowers to prevent the leaf yellowing (Ferrante et al., 2009). The reduction of functional gibberellins content or the conjugation of them with glucose (inactivation) induced leaf yellowing in several sensitive species. The exogenous applications are able to delay senescence and reduce ethylene biosynthesis. In cut stock flowers, the gibberellin 3 (GA_3_) applications did not enhance the ethylene biosynthesis, but strongly increased ethylene production, combining with thidiazuron (TDZ) (Ferrante et al., 2009). However, leaf yellowing was not affected by the ethylene production. This showed that the tissues were insensitive to ethylene because the leaves probably were not ready to senesce. However, further research should be taken into consideration to reveal the exact role of both the hormones in leaf senescence.

#### Ethylene and Abscisic Acid

Abscisic acid is considered a leaf senescence inducer and its exogenous applications lead to leaf senescence in mature leaves of different crops. The ABA content increased during leaf senescence and the exogenous treatment with ABA accelerate the leaf senescence (Oh et al., 1997; Yang et al., 2003). The *saul1* mutant (Senescence-Associated E3 Ubiquitin Ligase 1) naturally exhibited an accelerated leaf senescence phenotype with an increase of the ABA level, providing genetic evidence of the ABA signaling role during leaf senescence (Raab et al., 2009). The use of *ore* mutants which showed a delayed leaf senescence phenotypes in the following treatments with ABA and ACC suggested that ORE1, ORE3, and ORE9 were required for the proper progression of leaf senescence mediated by both ABA and ethylene (Kim et al., 2011).

## Flower Development

### Ethylene

The floral transition is a major progress in the plant’s life cycle that signals the onset of conditions favorable for reproductive success (Simpson and Dean, 2002). The exact timing of flowering can be controlled by the plant-environment interaction and endogenous developmental competence of plants to flower, which allows the transition from the vegetative phase to a reproductive phase (Lin et al., 2009). Changes in the levels of ethylene influence the genetic circuits that integrate different signals for the regulation of flowering timing. In *Arabidopsis*, through the growth comparison of ethylene-related mutants, *eto1*, *etr1*, *ein2-1* and *ein3-1*, with the wild-type (WT), the regulatory role of ethylene in the transition from vegetative to reproductive growth in *Arabidopsis* was discovered (Ogawara et al., 2003). The ethylene-overproducing mutant *eto1*, produces an excessive amount of ethylene (Guzman and Ecker, 1990) by affecting the post-transcriptional regulation of a key enzyme of ethylene biosynthesis, the 1-aminocyclopropane-1-carboxylic acid synthase (ACS) (Woeste et al., 1999), whereas the *ein2-1*, *ein3-1* and *etr1* mutants are insensitive or had a reduced sensitivity to ethylene (Bleecker et al., 1988; Guzman and Ecker, 1990). These perturbations in the ethylene signaling may flow large or less amount of ethylene signal respectively, into the hormonal pathway leading to an early- or late-flowering phenotype compared to WT (Ogawara et al., 2003). However, the effects of ethylene in the regulation of flower transition appear complex. In fact, the mutation of Ser/Thr kinases *CTR1* (At*ctr1*), which is the key negative regulator of ethylene signaling and the ACC-treated WT showed delayed flowering, indicating that ethylene inhibited flowering in *Arabidopsis* (Achard et al., 2007). In addition, contrasting roles of ethylene have been noticed in rice (*Oryza sativa*). Ethylene promotes a reproductive transition in rice through the activity of its receptor protein *OsETR2* (Wuriyanghan et al., 2009). In this study, the overexpression of *OsETR2* reduced ethylene sensitivity and delayed flower development, whereas the knockdown mutations of *OsETR2*, *OsETR3*, and *OsERS2* exhibited enhanced ethylene sensitivity and early flowering. Conversely, flowering time was delayed in *Osctr2* loss-of-function and *35S:OsCTR2* transgenic lines, indicating that ethylene represses the floral transition in rice (Wang Q. et al., 2013). These evidences suggest that ethylene signaling delays flowering in both rice and *Arabidopsis*. On the other hand, exogenous ethylene, or ethephon, has been widely used to induce flowering of Bromeliads, such as *Ananas comosus* and *Aechmea fasciata*, as well as early sprouting, early flowering and formation of more flowers per inflorescence in dormant corms of common triteleia (*Triteleia laxa*; Han et al., 1989). Furthermore, an inhibitor of ethylene biosynthesis, amino vinylglycine (AVG), can delay the natural flowering of pineapple (Kuan et al., 2005). Trusov and Botella (2006) proposed that the pineapple flowering is triggered by a small burst of ethylene production in the meristem in response to environmental cues through the induction of ACC synthase gene *AcACS2*. Moreover, the silencing of this gene *AcACS2* has been shown to delay flowering in pineapple. Overall, these results are consistent with ethylene having a fundamental role in flower development and may be related to different endogenous and external cues, which affected the ethylene signaling components.

Flower development occurs through a series of sequential steps required for the cell proliferation proper regulation, expansion, and the reproductive tissue development. The expression of ethylene biosynthesis genes seems to be linked to the formation of particular flower tissues. In tobacco, ACC oxidase (*ACO*) gene was expressed in early developing stigma, style, and ovary (De Martinis and Mariani, 1999). In tomato, *LeACO1*,*2*,*3*, and *4* and *LeACS1A* transcripts were detected in pistils (Llop-Tous et al., 2000). In the China rose (*Hibiscus rosa-sinensis*), *ACS* and *ACO* were found to be specifically expressed in developing style–stigma plus stamen and ovary tissues (Trivellini et al., 2011a). Similar evidence has been reported in carnation (*Dianthus caryophyllus*) and petunia (Tang et al., 1994; Jones, 2002), and may indicate that ethylene plays a role in the reproductive process during the development of flowers. Ethylene receptors are involved in reproductive organ development. In China rose *HrsETR* and *HrsERS* transcript levels were differentially expressed in the bud flower stage in style-stigma plus stamen, petals and ovary with different temporal patterns suggesting a possible tissue-specific role (Trivellini et al., 2011a). In pineapple, the expression of the ethylene receptors *AcERS1a*, *AcERS1b*, *AcETR2a*, and *AcETR2b* was higher in bract primordia and flower primordia ethephon-treated (Li et al., 2016), suggesting an important role during inflorescence development because ethylene induces pineapple flowering. In *Arabidopsis*, *ETR2* receptor was developmentally regulated in the inflorescence, floral meristems, and developing petals and ovules (Sakai et al., 1998).

Flower development occurs with the specification of floral identity in shoot meristem and then floral organ primordial initiates and rises to the formation of sepal, petal, stamen, carpel, and ovule. The development of floral organ is controlled by homeotic genes during reproductive phase. Each of these steps involves elaborate networks of factors that regulate floral morphogenesis. A potential genetic network involving ethylene as a regulator of flower development and homeotic genes has been emerging. Flower locus protein T (FT) is the major component of the mobile flower-promoting signal florigen and promotes the transition from vegetative growth to flowering in plants, ensuring the regulation of floral meristem identity genes such as APETALA (*AP*) and *LEAFY* (Krizek and Fletcher, 2005). In silver vase (*A. asciata*) using a comparative global transcriptome profile between adult and juvenile plants under ethylene, treatment was reported of the downregulation of TARGET OF EAT 1 (*TOE1*) and *TOE3*, belonging to *AP2*-like transcription factors, in adult plants (Li et al., 2015). These results suggest that the *AP2* family genes, such as *TOE1* and *TOE3* acting as repressors of *FT*, may participate in the induction of flowering by ethylene. In tomato, the ectopic expression of *LeHB-1* was reported to disrupt flower development, suggesting a critical role in floral organogenesis (Lin et al., 2008). *LeHB-1* encodes a class-I HD-Zip protein that binds to the promoter of *LeACO1*, involved in the regulation of tomato floral organogenesis, carpel development and ripening (Lin et al., 2008). A novel tomato mutant altered in the formation of floral organs, called unfinished flower development (*ufd*), showed higher hormone contents, particularly the ethylene precursor ACC compared to wild type (Poyatos-Pertíñez et al., 2016). Moreover, the global transcriptome profile showed that several MADS-box genes regulating floral identity as well as genes related to ethylene response were affected in *ufd* mutant inflorescences. These results suggest that ethylene signaling may interact with the development of flower primordia and *UFD* may have a key function as a positive regulator of floral organ identity and growth genes, together with hormonal signaling pathways.

### Interaction between Ethylene and Other Hormones during Flower Development

The present section gives an insight into the interaction of ethylene with other hormones during flower development.

#### Ethylene and Auxin

Auxins may influence flowering in plants by affecting ethylene evolution. In a classical study, Burg and Burg (1966) reported that auxin-induced flowering in pineapple by stimulating ethylene formation. Treatment of pineapple plants with naphthalene acetic acid (NAA) enhanced ethylene levels. However, this is an exceptional case, and ethylene generally inhibits flowering in many plant species, including *Arabidopsis* and pharbitis (*Ipomoea nil*, synonym *Pharbitis nil*) (Achard et al., 2007; Kaęsy et al., 2008, 2010). Achard et al. (2007) showed that in *Arabidopsis* grown under short-day (SD) conditions, ethylene delayed flowering in a DELLA-dependent manner and the inhibitory effect of auxins on flowering in pharbitis was caused by the induction of ethylene production (Kaęsy et al., 2008). Both the induction and inhibition of flowering have been reported by IAA, inhibition in SD plants cultivated under an inductive photoperiod, whereas stimulation in long-day (LD) plants under non-inductive conditions (Kulikowska-Gulewska et al., 1995; Wijayanti et al., 1997).

#### Ethylene and Abscisic Acid

Ethylene acts as a strong inhibitor of flowering in SD plants but only when it is applied in the second half of the inductive night (Kaęsy et al., 2008; Wilmowicz et al., 2008). ABA plays an important role in the photoperiodic induction of flowering in pharbitis seedlings, and the inhibitory effect of ethylene on pharbitis flowering inhibition may depend on its influence on the ABA level. The inhibition of flowering was observed when ABA was applied just before or at the beginning of a 16-h-long dark period (Wilmowicz et al., 2014). Moreover, the application of AVG partially reversed the inhibitory effect of ABA on flowering, suggesting that ABA influenced ethylene production which directly inhibited flowering. Thus, ABA could affect flowering indirectly by modifying other hormones. In Arabidopsis, ABA-deficient mutants *aba2-1* and *aba1-6* have a late flowering phenotype (Riboni et al., 2013) and the level of floral suppressing hormone ethylene have been shown to increase in *aba* mutant (LeNoble et al., 2004), and this condition may contribute to their late flowering phenotype.

#### Ethylene and Gibberellins

Various GAs, such as GA_32_ and 2,2-dimethyl G_4_, are especially florigenic when applied to non-induced Darnel ryegrass (*Lolium temulentum*) plants (Pharis et al., 1987). The treatment of GA to the foliar bud of japtropha (*Jatropha curcas*) increased the number of female flowers and fastened the flower development due to an increased endogenous level of GA and auxin. In contrast, ethrel (ethylene source) treatment decreased flower development due to the decreased endogenous level of auxin, while GA treatment significantly increased it (Makwana and Robin, 2013). Lee et al. (1998) suggested that the rhythm of bioactive GA production might play a role in the initiation of flowering. The pulses of GAs (especially GA_1_) may have different effects on floral initiation according to the time of day that they occur. The diurnal rhythm might be one way by which the absence of phytochrome B causes early flowering in 58M (phytochrome B null mutant) under most photoperiods. The expression of key oxidase genes in the biosynthesis of gibberellin, gibberellin 20 oxidase 2 (*GA20OX2*) is high in flowers and siliques, as is the expression *of GA20OX3* (Phillips et al., 1995; Dugardeyn et al., 2008). However, Mitchum et al. (2006) reported lower levels of *GA3OX2* during the later stages of development (in stems, flowers, and siliques). The GA-deficient mutant, *gal-3*, which is severely defective in ent-kaurene production (Zeevaart and Talon, 1992) flowers later than the Thale cress (*Landsberg erecta*) wild type in a long day but is totally unable to flower in SD unless treated with exogenous GA_3_ (Wilson et al., 1992). Although it is quite apparent that GA governs flowering in plants, however, its independence of ethylene is also an important question to be addressed.

The growth of plants in the presence of an ethylene precursor (ACC) or in an ethylene-enriched atmosphere delayed WT flowering (Achard et al., 2006). These findings were the basis for the current model for integration of the ethylene and GA–DELLA signaling pathways in the regulation of the floral transition (Achard et al., 2007). Previous analyses have shown that *CTR1* is the major negative regulator of ethylene signaling (Kieber et al., 1993). A study of Achard et al. (2007) found that the *ctr1–1* loss-of-function mutation confers late flowering under any photoperiod. Moreover, the ethylene-mediated inhibition of *CTR1* activity resulted in a reduction in bioactive GA levels, causing increased accumulation of DELLAs, a family of nuclear growth repressor proteins (Achard et al., 2007). DELLAs repress plant growth, whereas GA promotes growth via the mitigation of DELLA-mediated growth inhibition (King et al., 2001). Accumulation of DELLAs, in turn, delayed the initiation of the floral transition by repressing the up-regulation of the floral meristem identity genes *LEAFY (LFY)* and *SUPPRESSOR OF OVEREXPRESSION OF CONSTANS1 (SOC1)* (Achard et al., 2007). These results indicate that ethylene affects the GA biosynthesis and its interaction with GA governs the stability of DELLA proteins and hence flowering.

Transcript meta-analysis suggests that applying exogenous ethylene to plants represses the expression of GA metabolism genes. Conversely, upon treatment with GAs, the expression of some ethylene synthesis genes is up-regulated. At reduced ethylene levels, the growth of *gai-t6 rga-24* double loss-of-function mutants is more resistant to the effects of ACC than the wild type. Furthermore, in WT seedlings, GA treatment can substantially overcome the ACC-induced inhibition of root growth (Achard et al., 2003). Ethylene up-and down-regulates different GA biosynthesis and catabolism genes in *Arabidopsis* seedlings (Vandenbussche et al., 2007).

## Flower Senescence

The life of flowers is genetically determined due to their role in sexual reproduction and fertilization, and the maintenance of floral structure has a considerable cost in terms of respiratory energy, nutrients, and water loss (Stead, 1992; Jones et al., 2009). The flowers are therefore programmed to senesce after pollination or when the stigma is no longer receptive. In fact, young and mature petals are sinks, and only after pollination, when fertilization and fruit set are accomplished, a controlled senescence program allows important nutrients to be salvaged from dying tissue, from the petal to the developing ovary or transported to other sink tissues (i.e., young leaves), before flower death occurs (Rogers, 2013; Rogers and Munné-Bosch, 2016).

Flower senescence involves an ordered set of events coordinated at tissue and cellular level that can be regulated by endogenous signals, such as plant hormones, and by environmental factors, such as temperature, nutrients, light, and pathogen attack. All major plant hormones have been reported to affect flower senescence, with ethylene, jasmonic acid, salicylic acid (SA), ABA, and brassinosteroids as inducers and with cytokinins, GA, and auxin as inhibitors (Reid and Chen, 2008).

Ethylene is known to be a key player of plant aging, including fruit ripening, and flower and leaf senescence (Abeles et al., 1992). Ethylene in flower petals is involved in the inhibition of cell expansion through the regulation of water channel proteins (aquaporin) that facilitate the passage of water through biological membranes (Ma et al., 2008). The crucial role of aquaporins in flower development suggests that cellular collapse during the flower aging process might be regulated by transcellular and the transmembrane water transport which are important for motor cell dynamics. This condition might be supported by the massive transcriptional regulation of over 300 genes encoding for aquaporins among different flower developmental stages, from anthesis to senescence, in China rose (Trivellini et al., 2016).

A large number of flowers are affected by ethylene, but sensitivity to ethylene varies according to species and cultivars (Van Doorn, 2001). In many ethylene sensitive species, pollination triggers senescence leading to a climacteric rise in ethylene production, which becomes autocatalytic and coordinates cellular events among and within the different floral tissues, leading to wilt, fade, and abscise (Woltering and Van Doorn, 1988). The use of pharmacological treatments affect at different levels the ethylene signaling pathway, [i.e., AVG and AOA which affect the ACS enzyme, or, silver thio sulfate (STS) and 1-MCP which prevent ethylene to bind to its receptor, thus modulating the tissue sensitivity to the hormone], reveals collectively an intricate network of interactions as exemplified by numerous studies of senescence in flowers reviewed in Ferrante et al. (2015). For example, the exogenous application of ethylene or its biosynthetic precursor such as ACC accelerates corolla senescence in China rose flowers (Trivellini et al., 2011a). On the other hand, senescence can significantly delayed the treatment of flowers with inhibitors of ethylene biosynthesis, such as AOA (Trivellini et al., 2011b), or action, such as 1-MCP (Trivellini et al., 2011a).

Acid synthase catalyzes the synthesis of ACC, which is directly converted into ethylene by the ACO (Wang et al., 2002). Expression of *ACS* and *ACO* genes increased and are often coordinately regulated during flower senescence (Trivellini et al., 2011a; Ichimura and Niki, 2014; Tanase et al., 2015). Their suppression by antisense technology has been successful in prolonging floral display life. The down-regulation of the *ACS* and *ACO* genes in carnation reduced ethylene production and was effective in delaying floral senescence (Savin et al., 1995; Kiss et al., 2000). The antisense transformations of ethylene biosynthetic genes have been successfully attempted in other ornamental species including petunia (Huang et al., 2007), torenia (Torenia fournieri; Aida et al., 1998), and Christmas begonia (*Begonia x cheimantha*; Hvoslef-Eide et al., 1995). ACS is the rate-limiting enzyme of ethylene biosynthesis in plants (Wang et al., 2002), and its activity regulation may involve post-transcriptional regulation through its degradation (Wang et al., 2004). In *Arabidopsis* the *ETHYLENE-OVERPRODUCER1*-like (*EOL1*), negatively regulates ethylene biosynthesis (Wang et al., 2004) and in petunia the VIGS-mediated suppression of *PhEOL1* accelerated the senescence of flowers and increased ethylene production in corollas (Liu et al., 2016).

**FIGURE 1:**
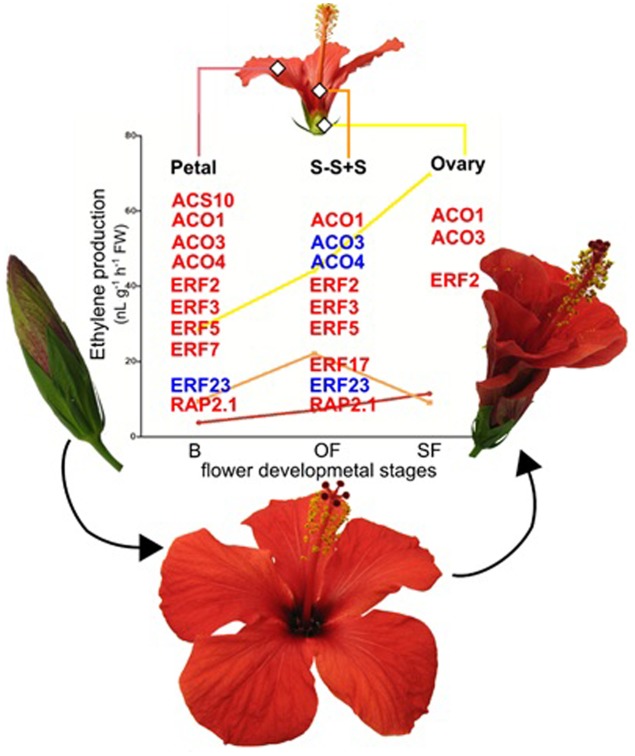
**Schematic representation of flower senescence in *H. rosa-sinesis* L.**. Representative flowers in bud (B), open (OF) and senescent (SF) flower stages. Ethylene changes in different flower organs, petal (pink line bar), style-stigma plus stamens (S-S+S; orange line bar) and ovary (yellow line bar). Data from Trivellini et al. (2011b). Ethylene biosynthetic (ACS and ACOs) and ethylene response factor genes (ERFs) differentially expressed in senescence flower organs. Red and blue indicate up-regulation and down-regulation, respectively. Data from Trivellini et al. (2016).

A positive feedback regulation, in senescing the China rose flowers through an increase in ethylene production among the different flower organs (Trivellini et al., 2011a,b) with the activation of *ACS* and/or *ACO* (Trivellini et al., 2016) is shown in **Figure 1**. Recently, the global transcriptome profiling of China rose reveals that the senescence is caused by the enhancement of signals that would naturally occur via transcriptional upregulation of the ethylene biosynthetic pathway during aging (Trivellini et al., 2016). In addition to the transcripts associated with biosynthetic genes (*ACO* and *ACS*), also the ethylene response factors (*ERFs*) were differentially regulated among flower tissues during senescence (**Figure 1**).

Ethylene perception mechanism and its signaling pathway are based on the presence of its receptors, which are essential to carry on the aging process (Kieber et al., 1993; Yoo et al., 2009; An et al., 2010; Ju et al., 2012; Ju et al., 2015). The alteration of ethylene signaling by transformations of several ornamental species (such as campanula, dianthus and kalanchoe) with the *ETR1* mutated gene under control of the flower-specific promoters resulted in plants with considerably higher ethylene tolerance and a better flower longevity (Gubrium et al., 2000; Sriskandarajah et al., 2007; Sanikhani et al., 2008). Moreover, transgenic petunia plants with reduced *PhEIN2* expression exhibited significant delays in flower senescence (Shibuya et al., 2004). And in *Arabidopsis*, a mutation in the *CTR1* gene causes a constitutive ethylene response and early senescence and abscission of the flowers (Huang et al., 2003) suggesting again a central role of ethylene in the promotion of flower senescence.

The role of ethylene receptors in the regulation of ethylene signaling is subject to modification from various proteins such as RESPONSIVE TO ANTAGONIST1 (RAN1; Binder et al., 2010), REVERSION TO ETHYLENE SENSITIVITY1 (RTE1; Dong et al., 2010; Qiu et al., 2012) and RTE1-HOMOLOG (RTH; Stepanova and Alonso, 2009). In petunia, no interaction was detected between *cEYFP–PhGRL2*, (homolog to *Arabidopsis RTE1* and the tomato GREEN RIPE, *SlGR*) and *nEYFP–PhETR1-3a* (closest homolog to the *Arabidopsis ETR1*) (Tan et al., 2014). By co-immunoprecipitation analysis, these authors demonstrated that *PhGRL2* interacts with *PhACO1*. Moreover, the suppression of *PhGRL2* by VIGS system conferred an accelerated flower senescence phenotype with enhanced ethylene production, and when *PhGRL2* was transiently overexpressed in petunia buds, the ethylene production was reduced and the longevity of flowers treated with *35Spro:PhGRL2* was significantly prolonged. These results raised the possibility that *PhGRL2* directly negatively regulates ACO activity.

EIN3-regulated genes trigger a diverse array of ethylene responses (Solano et al., 1998; Fujimoto et al., 2000; Potuschak et al., 2003; Yoo et al., 2008). In the petunia flowers, expression profiles of the *ETHYLENE RESPONSE FACTOR* (*ERF*) transcription factor family genes were studied in detail (Liu et al., 2011) and these transcription factors appear to be associated with corolla senescence. Recently, silencing an *ERF* petunia transcription factor homeodomain-leucine zipper protein (*PhHD-Zip*) dramatically reduced ethylene production and the abundance of transcripts of genes involved in ethylene (*ACS*, *ACO*) and led to an increase in flower longevity (Chang et al., 2014).

The dynamic activation of transcription factors during flower senescence is a key mechanism that controls the age-dependent expression of several senescence-related genes. These transcription factors, in turn, regulate the expression levels of various genes that may influence the ethylene pathway indirectly (Olsen et al., 2015). The MADS box genes are transcription factors that contain the conserved MADS domain that is responsible for DNA binding (Immink et al., 2002). A delay in senescence and flower abscission has been observed in *35S:AGL15* and *35S:AGL18 Arabidopsis* overexpressing plants (Fernandez et al., 2000; Adamczyk et al., 2007). The ectopic expression of a MADS box gene *FOREVER YOUNG FLOWER* (*FYF*) caused a significant delay of senescence and a deficiency of abscission in flowers of transgenic *Arabidopsis* plants by the down-regulation of *ETHYLENE RESPONSE DNA-BINDING FACTOR 1* (*EDF1*) and *EDF2*, downstream genes in the ethylene response (Chen et al., 2011) and by activating an *ERF* gene (Chen et al., 2015) suggesting a role for *FYF* in regulating senescence/abscission by suppressing the ethylene response.

### Interaction between Ethylene and Other Hormones during Flower Senescence

In addition to studies which describe the influence of the individual ethylene hormone on flower senescence, there are also reports that describe the importance of hormonal interactions.

#### Ethylene and Cytokinins

Previous studies have shown that either exogenous application (Taverner et al., 1999) or increased exogenous production of cytokinins in transgenic lines overexpressing a senescence-associated gene (*SAG12*)-specific promoter for driving the expression of the isopentenyl transferase gene (*SAG12-IPT*) delays senescence (Xu et al., 2010). The overproduction of cytokinins in petunia flowers transformed with *P*-S*AG12- IPT* has been reported to delay corolla senescence and decrease sensitivity to ethylene (Chang et al., 2003). The *PSAG12-IPT* gene was also transferred to a miniature rose, as the first woody species to be transformed with *SAG12-IPT* system, resulting in increased ethylene tolerance due to specific up-regulation of the *IPT* gene under senescence promoting conditions (Zakizadeh et al., 2013). An increase in ethylene, in petunia flowers exogenously treated with cytokinin, was found during senescence, and the lack of a negative effect can be explained considering the expression of the ethylene receptors was down-regulated by treatment with BA (Trivellini et al., 2015). Similarly, the application of thidiazuron, a cytokinin-like compound, enhanced ethylene production but simultaneously extended vase life by inhibiting leaf yellowing in cut stock flowers (Ferrante et al., 2012). These results suggest that despite the enhanced ethylene production, flowers that accumulated cytokinins showed an increased flower longevity. In Iris (*Iris germanica*), the flower senescence is apparently not regulated by endogenous ethylene, auxins, GA or SA (Van Doorn et al., 2013). In contrast, exogenous cytokinins delayed senescence, suggesting they might play a role in the regulation of the time of senescence (Van Doorn et al., 2013).

#### Ethylene and Gibberellins

The *HD–Zip I* transcription factors are unique to plants and have been reported to be involved in various plant development responses, including flower senescence (Xu et al., 2007). In rose, ABA or ethylene treatment clearly accelerated petal senescence, while the application of the gibberellin GA_3_ delayed the process and silencing of *RhHB1* delayed the ABA- or ethylene-mediated senescence in the rose petals (Lü et al., 2014). Moreover, the silencing of the key regulatory enzyme in the GA biosynthetic pathway, *RhGA20ox1* accelerated the senescence in rose petals. Thus, *RhHB1* mediates the antagonistic effect of GAs on ABA and ethylene during rose petal senescence, and the induction of petal senescence by ABA or ethylene operates through a *RhHB1-RhGA20ox1* regulatory checkpoint. Another recent study suggests that a reduction in the bioactive GA content enhances the ethylene-mediated flower senescence (Yin et al., 2015). In this study, the overexpression of a basic helix-loop-helix (*bHLH*) transcription factor, *PhFBH4*, increased the abundance of transcripts of ethylene biosynthesis genes and also increased ethylene production. Moreover, the increased expression of the GA metabolic gene *GA2ox3* in *PhFBH4-OX* transgenic plants would raise bioactive GAs content, while silencing *PhFBH4* would reduce their levels (Yin et al., 2015). Another study reported that the transcriptome changes associated with delayed flower senescence on transgenic petunia by inducing the expression of *etr1-1*, down-regulated genes involved in gibberellin biosynthesis, response to gibberellins stimulus, and ethylene biosynthesis, at different time points (Wang H. et al., 2013).

#### Ethylene and Abscisic Acid

Similarly to the ethylene, ABA accumulation accelerates the senescence of cut flowers and flowering potted plants (Ferrante et al., 2015). In rose, ABA was reported to increase the sensitivity of flowers to ethylene, as the gene expression of some ethylene receptors increased after exogenous ABA treatment (Muller et al., 2000). On the other hand, ABA negatively affected the ethylene biosynthetic pathway and in hibiscus (*Hibiscus rosa-sinensis*) tissue sensitivity in all flower tissues, reducing the transcript abundance of *HrsACS*, *HrsACO*, *HrsETR*, and *HrsERS* when exogenously applied (Trivellini et al., 2011a). The over-expression of *PhHD-Zip* accelerated petunia flower senescence and this condition is another example highlighting the interaction of different hormones (Chang et al., 2014). In fact, *PhHD-Zip* transcript abundance in petunia flowers was increased by the application of hormones (ethylene, ABA) and the transcript abundance of 9-*cis*-epoxycarotenoid dioxygenase (*NCED*), a key enzyme in the ABA biosynthesis pathway, was in contrast in *PhHD-Zip* silenced flowers. These results suggest that *PhHD-Zip* plays an important role in regulating petunia flower senescence. Moreover, a transcriptome study reported that several genes involved in ABA biosynthesis, catabolism, and signaling pathways were induced by exogenous cytokinins (BA) treatment (Trivellini et al., 2015). In the experiment reported by Chang et al. (2003), transgenic lines of petunia overexpressing *IPT* gene, displayed a lower endogenous ABA level compared to the wild type, and this condition was confirmed by BA treatment which delayed senescence by lowering the ABA content with a higher ethylene production (Trivellini et al., 2015). These results suggest that in addition to the ethylene pathway, the cytokinins seem to be strongly involved in the regulation of ABA biosynthesis and its degradation in flower tissues, thus ABA plays a primary role in petunia flower senescence.

## Fruit Ripening

### Ethylene

The fruit is the development of the ovary after the fertilization and protects the seeds until complete maturation. The seeds represent the germ plasm of the plants and are responsible for the dissemination of the species. From an ecological point of view, fruits during the unripe stage represent an organ that must be protected from insects or frugivores. A fruit must be unattractive and its green color allows the camouflage itself with leaves. The ripening of fruits is a unique coordination of various biochemical and developmental pathways regulated by ethylene, which affects color, texture, nutritional quality and aroma of fruits (Barry and Giovannoni, 2007).

During ripening in climacteric fruits, the ethylene regulates firmness and color changes involving chlorophyll reduction, increase in carotenoids or anthocyanins, sugars, and biosynthesis of volatile organic compounds (VOCs).

**FIGURE 2:**
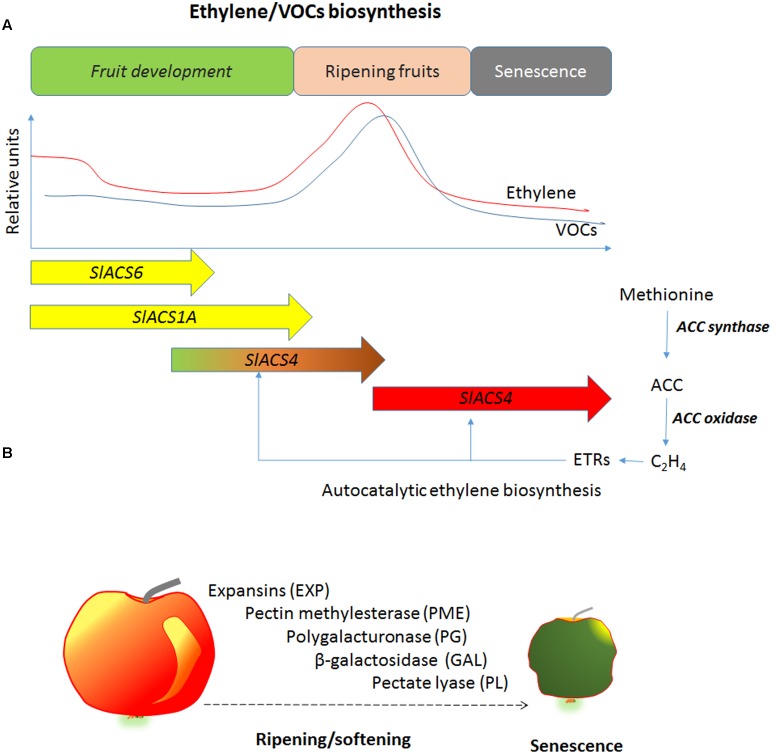
**(A)** Schematic and simplified ethylene and VOCs biosynthesis during fruit development. *SlACS* (*Solanum lycopersicum* ACC synthase) families are differentially expressed during fruit development. VOCs biosynthesis derive from different pathways such as phenylpropanoids, fatty acid, and carotenoids degradation. **(B)** The main enzymes involved in cell wall degradation during fruit ripening and senescence. The action of these enzymes induces loss of firmness and softening.

Ethylene is tightly correlated with the VOCs biosynthesis, which increases in ripe fruit and enhances the attraction of frugivores. The inhibition of ethylene biosynthesis reduces production of VOCs and reduces the aroma of fruits (**Figure 2**). It has been found that transgenic apples expressing antisense genes for ACS or ACO produced lower VOCs and in particular, the strongest reduction was observed in the esters, which were 3–4 fold lower compared with WT (Dandekar et al., 2004). The same behavior has been observed in ACO antisense melon (*Cucumis melo*) fruits, the esters were inhibited and were 60–85% less than the control plants (Bauchot et al., 1998; Flores et al., 2002). The exogenous application of ethylene reconverted the VOCs evolution. This result indicates that ethylene inhibits the key steps of volatile biosynthesis. The study with the application of 1-MCP or AVG demonstrated that ethylene regulates VOCs biosynthesis directly through the pathway of volatile biosynthesis and indirectly through the ethylene perception. In fact, apricots (*Prunus armeniaca*) treated with ethylene biosynthesis inhibitor, such as AVG, strongly reduced the VOCs biosynthesis, while the 1-MCP, an ethylene action inhibitor, enhanced the evolution of aldehydes (Valdes et al., 2009).

The relationship between fruit ripening and ethylene/respiration pattern allows the classification of fruits as climacteric or non-climacteric. In climacteric fruits, ethylene biosynthesis increases and shows a peak corresponding to respiration pattern, while in non-climacteric fruits the ethylene declines with fruit ripening and senescence.

The tomato has been used as a model plant for studying the role of ethylene in fruit ripening. The transition from unripe to ripe fruit induces several biochemical changes that involve ethylene biosynthesis and perception. Unripe fruits produce a low amount of ethylene and are insensitive to exogenous ethylene. Hence, ethylene treatments do not induce the fruit ripening (system 1). At the beginning of ripening, ethylene production increases and induces an increase of autocatalytic biosynthesis. These fruits, in this development stage, if exposed to exogenous ethylene show a burst of ethylene production and ripen faster (system 2). These two systems are proposed to explain the auto-inhibitory effect of the ethylene during vegetative growth and the auto-stimulatory effect of the ethylene during ripening (Lelièvre et al., 1998). Fruits are classified in system 1 when they produce a low amount of ethylene and tissues are insensitive to exogenous ethylene (Alexander and Grierson, 2002). At this stage, ethylene biosynthesis is regulated by *ACS6* and *ACS1* genes. The delay of ethylene increase is the most common strategy used in post-harvest for prolonging the storage and increasing the shelf life. The inhibition of ethylene biosynthesis or action usually leads to an extension of shelf life of the climacteric fruits.

Ethylene regulates fruit ripening by affecting the *ACS* and *ACO* genes and the fruit specific polygalacturonase, involved in the depolymerization of cell wall pectin during ripening (Smith et al., 1988). It affects pectin methylesterase (PME), which provides accessibility to pectin by polygalacturonase and phytoene synthase responsible for the pigmentation of many fruits and flowers (Koch and Nevins, 1989; Fray and Grierson, 1993).

Cloned mRNAs that accumulate in the unripe tomato fruits exposed to exogenous ethylene were investigated through blot hybridization experiment. The expression of cloned genes was developmentally regulated by the ethylene during fruit ripening, with more mRNAs produced by these genes in ripe fruits than in unripe fruits and the increase in mRNA was repressed by norbornadiene, an ethylene action inhibitor (Lincoln et al., 1987).

Tomato ethylene receptor, *LeETR4* or *LeETR6*, played an important role in flowering and reduction in its expression level (Tieman et al., 2000; Kevany et al., 2007). Gene expression analysis of Never-ripe (*Nr*) and additional tomato receptor homologs indicated that *Nr* and *LeETR4* transcripts were most abundant in the ripen fruit tissues (Zhou et al., 1996; Lashbrook et al., 1998).

Alba et al. (2005) identified 869 genes, differentially expressed in developing tomato pericarp. A 37% of these differentially expressed genes showed altered expression patterns in the *Nr* mutant background. The mutation of the ethylene receptor *Nr*, which reduces ethylene sensitivity and inhibits ripening, also influenced fruit morphology, seed number, ascorbate accumulation, carotenoid biosynthesis, ethylene evolution, and the expression of many genes during fruit maturation, indicating that ethylene governed multiple aspects of development both prior and during fruit ripening in tomato (Alba et al., 2005). In tomato, the E8 gene plays a role in the negative regulation of ethylene biosynthesis through repression of ethylene signal transduction. The expression of the gene increased during ripening and its antisense repression resulted in an increased ethylene evolution but delayed ripening (Penarrubia et al., 1992).

### Interaction between Ethylene and Other Hormones during Fruit Ripening and Senescence

#### Ethylene and Auxin

The relationship between ethylene and auxin in the fruit development has been studied. Auxins are involved in fruit development and inhibit ripening (Brady, 1987). The exogenous application of auxins in different fruits delayed the senescence such as observed in Bartlett pears (*Pyrus communis*; Frenkel and Dyck, 1973), banana (*Musa acuminate*; Purgatto et al., 2001), peaches (Trainotti et al., 2007) and strawberry (*Fragaria ananassa*; Harpster et al., 1998). The application of auxin lowered the ethylene production in sliced apples (*Malus domestica*), if applied at pre-climacteric phase, while enhancing its biosynthesis at the climacteric stage (Lieberman et al., 1977). The transcription factor *AUXIN RESPONSE FACTOR 2A (ARF2A)* has been recognized as an auxin signaling component and was able to control ripening (Breitel et al., 2016). There exists a crosstalk between auxin and ethylene; and Bleecker and Kende (2000) pointed out that auxins can stimulate the biosynthesis of more climacteric ethylene through its inductive action on the expression of the key enzyme ACS (Abel and Theologis, 1996).

Ethylene and auxins are tightly related during fruit senescence. The free auxin increases during senescence and stimulates ethylene biosynthesis. Further studies are required to understand the ethylene sensitivity changes after 1-MCP treatment. The CTG134, a calcineurin B-like protein similar to GOLVEN (GLV) peptides, seems to shut down genes that are commonly repressed during ripening by ethylene and auxin treatments (Tadiello et al., 2016). The nature and transcriptional response of CTG134 led to discovering a rise in free auxin in the 1-MCP treated fruits.

#### Ethylene and Cytokinin

The exogenous application of cytokinins or compounds with cytokinins-like activity increased the sugar content of fruits and induced earlier ripening. Spray application on kiwi (*Actinidia deliciosa*) using *N*-(2-chloro-4-pyridyl)-*N*’-phenylurea (CPPU), a diphenylurea derivative cytokinin, increased the starch content and induced faster fruit development. Recent studies have shown that CPPU delayed the ethylene increase during fruit ripening and also delayed central placenta softening (Ainalidou et al., 2016). In avocado, the application of isopentenyl adenosine increased the ethylene and fruit ripening (Bower and Cutting, 1988). The studies regarding the role of cytokinins in the plant senescence are available in the literature, but the relationship between cytokinins and ethylene during fruit ripening and senescence has not yet completely been elucidated and needs further investigations.

#### Ethylene and Abscisic Acid

In tomato fruit, ABA biosynthesis occurs via carotenoids degradation pathways and the key enzyme is the 9-*cis*-epoxycarotenoid dioxygenase (NCED). The ABA content increases following the biosynthesis of carotenoids during ripening. These changes are associated with ripening and also with ethylene production. During fruit ripening, NCED gene expression occurs earlier than ACS or ACO which are also involved in the ethylene biosynthesis. The exogenous application of ABA increases ethylene biosynthesis (Mou et al., 2016). These results suggest that ABA can be a trigger for ethylene production and influence fruit ripening (Zhang et al., 2009). In banana fruit, ABA stimulates ripening independently from the ethylene. ABA application increases all hydrolases, which can enhance the softening, with exception to the polygalacturonase activity (Lohani et al., 2004). Recently, it has been reported that an ABA Stress Ripening (ASR) transcription factor acts as a downstream component of a common transduction pathway for ABA and sucrose signals during fruit ripening (Jia et al., 2016). Interestingly, these authors provide new insights into the regulatory mechanism underlying tomato fruit development and ripening with the ethylene involved in the downstream signal transduction of ABA and sucrose, as a negative regulator of ASR gene expression, which influenced the expression of several cell wall and ripening-related genes leading to fruit softening.

The relationship of other phytohormones such as ABA and GA with ethylene during fruit senescence needs to be elucidated.

## Fruit Senescence

### Ethylene and Enzymes Involved in Fruit Senescence

The loss of firmness or softening of fruits is a very important quality parameter. The softening is due to cell wall degradation induced from several enzymes that are synergistically activated. These enzymes are pectine methyl esterases, polygalacturonase, cellulase, galactosidases, pectate lyase (PL), xyloglucan transglucosylase/hydrolases, and expansins. Almost all these enzymes are encoded by multi-genes family, which regulates the spatial-temporal activation of these enzymes. Ethylene plays a crucial role in regulating these genes and enzymes during ripening and senescence. The cell wall degradation is facilitated by expansins that are proteins, which are involved in the enlargement of cell matrix. This phenomenon occurs during cell wall growth and disruption. The action of these enzymes has been found to be tightly associated with the fruit ripening and senescence (Civello et al., 1999). The expansins are tightly dependent on pH. The transcription of these enzymes is carried out by gene families, which have been isolated and characterized in several plant species. Different isoforms can provide the expansins action during plant growth and fruit senescence, linking the development stage with the activation of specific isoforms. During tomato ripening, *EXP1* was induced by ethylene exposure to concentrations higher than 1 μL L^-1^. The inhibition of ethylene biosynthesis also reduced and inhibited the *EXP1* gene expression (Rose et al., 1997). The activation of the expansin *EXP1* has also been shown in other climacteric fruits such as banana (Trivedi and Nath, 2004).

Pectin methylesterase is an enzyme activated before fruit ripening and catalyzes the de-esterification of pectin, by removing the methyl group C-6 of galacturonic acid and allows the polygalacturonase action. The PME has an important role during fruit senescence and cell wall degradation with loss of firmness. This enzyme is stimulated by ethylene and inhibited by ethylene inhibitors such as 1-MCP (El-Sharkawy et al., 2016). Exo- and endopolygalacturonase are involved in depolymerization of galacturonic acid, hydrolysis of bonds 1, 4 of homopolymers of α-D-galacturonic acid. This enzyme is activated after the action of PME and is also induced by ethylene. In antisense ACC synthase tomato, the exposure to ethylene rapidly increased transcript accumulation of the PG. The gene expression of PG was directly correlated with ethylene concentrations used (Sitrit and Bennett, 1998).

The β-galactosidase breaks bonds between β-(1,4)-galactans in the cell wall. This enzyme is involved in fruit softening by degrading the β-(1,4)-galactans pericarp cell wall (Eda et al., 2016). Transgenic tomato for antisense of β-galactosidase showed higher firmness at the red ripening stage (Smith et al., 2002). The inhibition of ethylene action in avocado using 1-MCP reduced β-galactosidase activity (Jeong and Huber, 2004). The exogenous ethylene application increased β-galactosidase activity in watermelon and the higher activities of these enzymes were observed in immature fruits (Karakurt and Huber, 2002). PL catalyzes the breakdown of 1-4 of α-D-galacturonic acid (Uluisik et al., 2016). Bananas treated with ethylene increased the activity of this enzyme, while the use of 1-MCP reduced its activity (Lohani et al., 2004). Analogous results were observed in mango treated with ethylene for inducing ripening or treated with 1-MCP for delaying ripening (Chourasia et al., 2006). The cell wall degrading enzymes is sequentially activated during ripening and senescence. Ethylene is one key regulator of these enzymes at transcriptional and post-transcriptional level (**Figures 2A,B**).

## Conclusion and Future Prospects

It may be summarized that ethylene plays a key role in plant growth and development. The action of ethylene in the growth and development may not be isolated. It triggers the network of signaling pathways and influences through the interaction with other phytohormones regulation of several processes. The understanding of the crosstalk between ethylene and other phytohormones in regulating growth and senescence could provide a promising strategy to manipulate the content of these hormones through molecular techniques in order to get specific plant responses.

During plant life, the transition from vegetative to reproductive stages and senescence is largely influenced by ethylene and its interplay with other plant hormones. This networking not only influences the ethylene concentration but also tissues sensitivity. There are few studies focusing on the molecular changes in plant tissues after the combined treatments of ethylene with other plant hormones. These studies should be extended to different organs and development stages to deeply understand the intricate network affecting relevant agronomic traits such as yield, longevity, and appearance (morphology). The discovery of new synergistic or antagonist relationships among ethylene and other hormones can have great potential to support cell division and differentiation processes during plant development, to enhance crop yield by delaying aging and prolong shelf-life of flowers and maintain the quality of climacteric fruits.

Moreover, the equilibrium between the ethylene biosynthesis and its perception influences the crop adaptability and performance under different stress conditions. It has been shown that other plant hormones can positively or negatively influence this equilibrium. The interplay of ethylene and plant hormones on plant performance should also be investigated at the post-translation level.

## Author Contributions

NI and MK wrote on the role of ethylene in leaf, flower and fruit growth and development and its interaction with other hormones in the process, together with the introduction. NK suggested the concept of the manuscript, wrote the abstract and looked over the whole manuscript order and language and contributed to the overall look of the manuscript. AFe, AT, and AFr wrote on the role of ethylene in leaf, flower and fruit senescence and its interaction with other hormones in the process.

## Conflict of Interest Statement

The authors declare that the research was conducted in the absence of any commercial or financial relationships that could be construed as a potential conflict of interest. The reviewer BVDP and handling Editor declared their shared affiliation, and the handling Editor states that the process nevertheless met the standards of a fair and objective review.
